# Changes in Muscle Fiber Growth and the Emergence of Muscle Myopathies in Current Commercial Meat Birds

**DOI:** 10.3390/ani16101553

**Published:** 2026-05-20

**Authors:** Md Raihanul Hoque, Casey Owens, Craig Coon, Pramir Maharjan

**Affiliations:** 1 Department of Poultry Science, Texas A and M University, College Station, TX 77843, USA; mhoque@tamu.edu; 2 Department of Poultry Science, University of Arkansas, Fayetteville, AR 72701, USA; cmowens@uark.edu (C.O.); ccoon@uark.edu (C.C.)

**Keywords:** broiler production, etiology, muscle myopathies, mitigation strategies

## Abstract

The rapid growth of broiler production efficiencies has been driven by selective breeding and optimized nutrition. However, these advancements have also introduced muscle myopathies such as wooden breast (WB), white striping (WS), and spaghetti meat (SM), which impair meat quality and cause major economic losses for the broiler industry. This review summarizes myopathy etiology, structural and physiological changes, detection methods, and mitigation strategies. It discusses factors such as accelerated muscle growth, poor circulation, oxidative stress, and metabolic imbalance, along with impacts on meat quality and industry economics. Current detection approaches range from visual and tactile assessments to advanced technologies like near-infrared spectroscopy and computer vision. Nutritional interventions have been evaluated as strategies to reduce the incidence of myopathies. The review emphasizes the need for further research to clarify underlying mechanisms and develop effective prevention and detection solutions.

## Simple Summary

This review explains how modern broiler chickens have been selectively bred and nutritionally managed to achieve rapid and efficient growth; however, these improvements have also led to muscle problems such as wooden breast, white striping, and spaghetti meat. These conditions reduce meat quality and cause financial losses for the poultry industry. The paper describes what causes these muscle disorders, how the muscle structure and function are changed, and how they affect meat quality. It also reviews different ways to identify these problems, from basic visual and hand inspection to more advanced tools, like near-infrared spectroscopy and computer-based imaging. In addition, the review discusses nutritional and feed-based strategies, including antioxidants and feed additives, that may help lower the occurrence of these muscle defects.

## 1. Introduction

Broiler meat production has expanded significantly over the years compared to other animal protein sources due to rising human health awareness, cultural preferences, production efficiency, and global population growth [1]. Selective breeding has primarily focused on improving growth rate, breast muscle yield, and feed efficiency, leading to a tenfold increase in breast muscle yield compared to broilers marketed in 1955 [2]. While these advancements have enhanced production efficiency, they have also introduced various meat quality defects, particularly wooden breast (WB), white striping (WS), and spaghetti meat (SM) [3,4,5].

These myopathies compromise meat quality by producing lower-grade breast filets, which reduces their appeal to consumers and negatively impacts the poultry industry’s profitability [6,7]. Affected meat is often sold at reduced prices or processed into lower-value products, resulting in financial losses. Based on a 23% breast yield of live weight, according to the National Chicken Council, the U.S. poultry industry generates approximately 12 billion pounds of breast meat annually. According to a conservative estimate, the prevalence of these myopathies can lead to a loss of over $200 million per year due to reduced yield and lost value if the product is downgraded or even discarded [8].

Despite their significant impact, the exact causes, detection methods, and prevention strategies for these myopathies remain unclear. Future research must focus on elucidating the underlying mechanisms, which are hypothesized to involve factors such as rapid muscle growth, impaired blood circulation, oxidative stress, and metabolic imbalances [9]. The complexity of these conditions necessitates further investigation to develop effective detection and prevention strategies that could mitigate economic losses and improve meat quality.

This review aims to provide a comprehensive overview of the present understanding of WB, WS, and SM, focusing on their proposed etiologies, detection methods, and potential prevention strategies. By accumulating current knowledge on the present direction of research on detection and mitigation methods, this review highlights critical gaps in knowledge and emphasizes the need for further studies to address these emerging challenges in modern broiler production.

## 2. Muscle Structure Changes over Time

In the last 50 years, extensive changes have occurred in broiler production (Table 1). According to Havenstein et al. [10], the reported hot carcass weight of unclassified male broilers on day 57 was only 539 g, whereas Arber Acre male broilers in 1991 had a hot carcass weight of 2367 g on day 57. Zuidhof et al. [11] mentioned that the FCR of meat broilers was 2.86 (day 0 to 57) in 1957, which decreased to 1.92 in 2005. Presently ROSS 308 has a final BW of 4310 g with an FCR of 1.79 on day 56 [12]. The biggest change is in the breast- and thigh-muscle yield of broilers.

Skeletal muscle originates from mesodermal precursor cells (myoblasts) that fuse to form multinucleated muscle fibers. These fibers contain myofibrils composed of repeating protein units called sarcomeres, which are the fundamental contractile structures of muscle [19]. Based on histochemical, biochemical, and ultrastructural characteristics, skeletal muscle fibers are generally classified into three types: slow-oxidative (type I), fast oxidative–glycolytic (type IIA), and fast glycolytic (type IIB) [20].

In broiler chickens, muscle composition is dominated by two primary fiber types: fast-twitch glycolytic (type IIB) and slow-twitch oxidative (type I). The pectoralis major (breast muscle) consists almost entirely of type IIB fibers, reflecting its role in rapid, short bursts of activity, whereas thigh muscles contain a mixed population, typically around 70% oxidative and 30% glycolytic fibers [21]. These fiber-type distributions are important determinants of muscle function, as well as meat quality attributes.

Muscle fiber number is largely established at hatch and is primarily influenced by genetic factors. Intensive genetic selection for rapid growth and increased breast yield in modern broilers has significantly altered muscle fiber characteristics [22,23]. Compared with slower-growing lines, modern fast-growing broilers exhibit greater muscle fiber hypertrophy, particularly in glycolytic fibers, with diameters increasing substantially during growth (e.g., from approximately 18 µm to 46 µm by day 42) [24,25,26,27]. In addition to hypertrophy, shifts in fiber-type composition have been reported, including an increased proportion of glycolytic fibers in the pectoralis major and a transition from oxidative (type I) to more glycolytic (type IIB) fibers in thigh muscles [2,25].

## 3. History and Features of Woody Breast (WB), White Striping (WS), and Spaghetti Meat (SM)

The intensive production of heavier broilers has been associated with an increased incidence of myodegeneration, which contributes to muscle myopathies and negatively affects the chemical composition and water-holding capacity of broiler meat [28,29]. The rapid growth rate of modern broilers may also impair thermoregulatory capacity, cation regulation, and glycolytic potential, further influencing meat quality. The most common muscle myopathies in modern broilers include WB, WS, and SM.

WB myopathy was first described by Sihvo et al. [3] in the pectoralis major muscle of broilers. Recent studies indicate that 30.8% of commercial broilers at 35 days of age exhibit WB symptoms [30]. Tijare et al. [31] reported that a research flock at 6 wk exhibited 48% mild, 28% moderate, and 20% severe WB. Che et al. [32] reported incidences of 70.5% for moderate WB and 11.8% for severe WB based on a population of 9200 filets from birds averaging 2.36 kg from multiple flocks.

**Figure 1:**
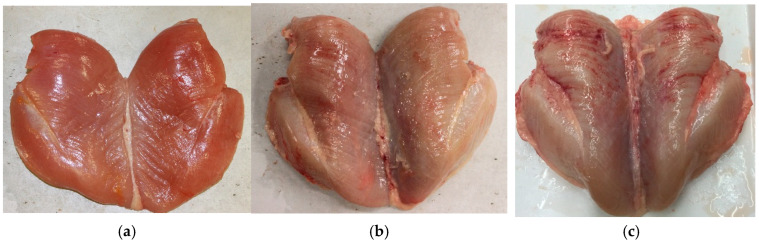
Severity of wooden breast (WB) myopathy is characterized by the relative hardness of broiler breast muscle. (**a**) Normal filet with WB score of 0, no hardness detected throughout the breast filet; (**b**) WB score of 2.5 and (**c**) WB score of 3. (**b**,**c**) Severely affected WB filets: hardness detected throughout cranial, medial regions with paler coloration of filet and the presence of exudate, hemorrhage and white fibrotic membrane (scoring system adopted from Tijare et al. [26]).

WB myopathy is primarily identified by the hardening of the breast muscle, which can be localized to the proximal region or extend throughout the entire muscle, depending on severity [30,31]. Other noticeable features include pale color, surface hemorrhages, and the presence of sterile exudate on the breast surface (Figure 1). Histologically, WB-affected muscles exhibit significant muscle fiber degeneration and regeneration, infiltration of immune cells, and increased accumulation of adipose and connective tissue [30]. A primary feature of WB is that the muscle fibers are replaced with an extracellular protein matrix like collagen [33]. Additionally, Zhu et al. [34] reported higher total collagen in WB-affected birds’ breast muscle compared to the normal broiler breast muscle. That study correlated higher collagen buildup with a higher susceptibility to WB in broilers. These pathological changes contribute to the stiff and rubbery texture of WB meat.

WS was initially observed in older hens and turkeys before being identified as a meat quality defect in broilers [35]. In modern intensive broiler production systems, up to 90% of chicken breast meat can be affected by WS [33]. Tijare et al. [31] reported that 96% of filets had some level of WS in a research flock at 6 wk. Che et al. [32] reported an incidence of 93.8% mild WS and 2.2% moderate WS in commercial flocks (average 2.36 kg).

**Figure 2:**
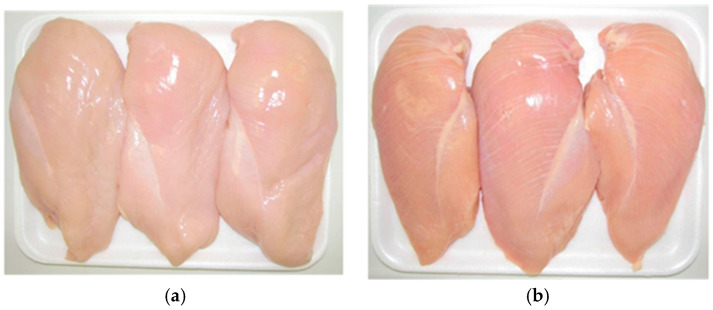
White striping (WS) in broiler breast meat. (**a**) Normal breast filet of WS score 0 without white striations; (**b**) severely affected WS filet of score 2.5 characterized by white striations across the filet with fat deposition. (Scoring system was adopted from Kuttappan et al. [37]).

WS is characterized by the presence of parallel white lines running along the breast muscle, which vary in thickness depending on severity (Figure 2). Although WS-affected meat does not exhibit the same degree of firmness as WB, its histological characteristics show similarities to WB. Histological analysis of WS reveals increased adipose tissue deposition, muscle fiber degeneration, and connective tissue regeneration [36,37,38]. The intramuscular fat synthesis and deposition in *Pectoralis major* muscle in myopathy -affected birds were observed by Petracci et al. [36].

### Spaghetti Meat (SM)

SM, previously referred to as “Mushy Breast,” was first recognized as a distinct broiler muscle defect in 2015, affecting the pectoralis major muscle [39]. Although its occurrence is lower compared to WB and WS, studies suggest that 10–21% of broiler breast meat may be affected [33]. However, Che et al. [32] reported 36.3% SM in flocks averaging 2.36 kg.

**Figure 3:**
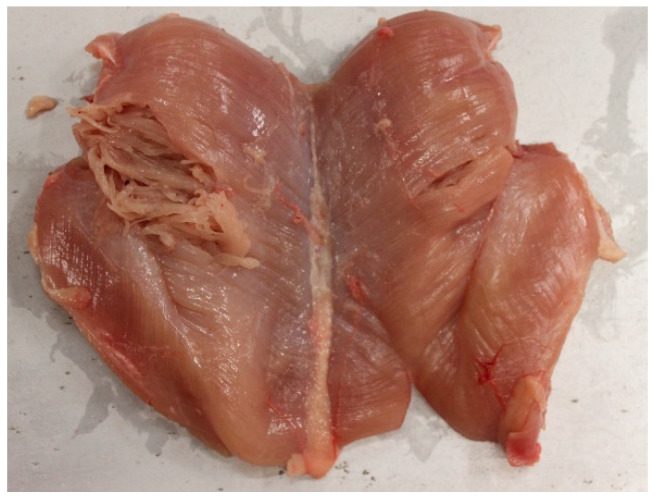
Spaghetti meat in broiler breast meat. The muscle loses integrity and shows separation of muscle fibers.

SM is distinguished by its soft, mushy texture, resembling spaghetti pasta (Figure 3) [40,41]. While SM shares some histological features with WB and WS, it also exhibits unique structural alterations. Common histological features of SM include nuclei internalization, fiber necrosis, muscle lysis, inflammation, and abnormal fiber regeneration [5]. However, key differences that set SM apart from WB and WS include rarefaction of the endomysial and perimysial connective tissue, the presence of small and thin fibers in newly formed connective tissues, and detachment of muscle fibers from each other [42]. These structural weaknesses contribute to the fragile and loose texture observed in SM-affected meat.

While these myopathies can occur individually, they often appear simultaneously, leading to overlapping meat quality defects. Che et al. [32] reported that 32.8% of filets exhibited WB, WS, and SM together, and approximately 50% exhibited WB and WS. Bowker et al. [40] also reported positive moderate correlations between WB and WS incidence. Despite their differences, WB, WS, and SM share common alterations in muscle texture, composition, and structural integrity. Their increasing prevalence in commercial broiler production highlights the need for further research into prevention strategies and management solutions.

## 4. Potential Etio-Physiology of Modern Myopathies

The genetic advancements in modern broilers have significantly enhanced production efficiency; however, they have also altered the structure of breast muscles. The protein turnover rate has improved significantly in the long run. In 1989, on day 38, the fractional synthesis rate (FSR) of protein in mixed muscle (including myofibrillar protein and mitochondrial protein) of the broiler was 15.8%/d, the fractional degradation rate (FDR) was 10.89%/d and the fractional accretion rate (FAR) was 4.9%/d [43]. In 2000, on day 38, the broiler FSR was 21%/d, the FDR was 14%/d and the FAR was about 7%/d [16]. The FSR has become 1.5 times higher in the meantime due to genetic and nutritional improvements. In myopathy-affected broilers, FDR can be higher than that of normal birds, which is an indication of rapid protein breakdown in the muscle [44]. These changes include increased muscle fiber diameter, length, and density, which, in turn, have reduced blood supply to the muscle tissue [45]. Genomic selection for faster growth rates and higher breast yields has been identified as a primary factor contributing to the development of muscle myopathies such as WB, WS, and SM [46]. While muscle myopathies are positively correlated with increased meat production, non-genetic factors also play a role. The heritability of WB, WS, and SM in commercial broilers has been reported at 0.024–0.097, 0.04–0.07, and 0.185–0.338, respectively [47].

The exact cause of broiler breast myopathies remains unclear, but several hypotheses have been proposed to explain their underlying mechanisms. Although the causes are not fully understood, WB, WS, and SM are believed to share a similar pathophysiological basis. One key hypothesis suggests that excessive breast muscle growth without a proportional improvement in the circulatory system leads to inadequate blood supply. Zambonelli et al. [48] suggested that modern breast myopathies have a multifactorial and complex etiology that might include muscle development, polysaccharide metabolism, proteoglycan synthesis, inflammation and calcium signaling pathway. Broiler breast muscle consists predominantly of type IIB glycolytic fibers, which rely on anaerobic metabolism and produce lactic acid as a byproduct [49]. Under normal conditions, the circulatory system transports lactic acid to the liver, where it is converted into glycogen and recirculated as an energy source [50]. However, the insufficient capillary network in the breast muscle compromises blood supply, oxygen availability, and waste removal, leading to muscle damage [43]. Reduced circulation also impairs nutrient transport and waste clearance, potentially causing phlebitis and perivascular lipid infiltration [51]. Another proposed etiology of breast myopathy is metabolic alteration. Type IIB muscle fibers contain a small volume (0.15 vs. 0.96 µm^3^) and small-sized (0.15 µm vs. 0.35 µm) mitochondria compared to the type I muscle [52]. Mitochondria are the primary organelles that produce ATP, and they are the primary targets of oxidative attack by reactive oxygen species (ROS). Due to the hypoxic condition in myopathy-affected muscles, mitochondria lose their cell integrity due to ROS attack and become inefficient at ATP production. This mitochondrial dysfunction contributes to inflammation and metabolic abnormalities [53,54]. Transcriptomic analysis has shown that the TCA cycle and glycolysis are reduced in the pectoralis major muscle in WB-affected birds [55]. In addition, the production of α-ketoglutarate, fumarate, and malate was observed, indicating these as intermediates of increased amino acid metabolism. These support the alteration of the metabolic pathway of energy source utilization and physiological action to reduce oxidative stress in muscle.

Collagen and lipid accumulation are additional events that occur in myopathy-affected broilers [16,56]. A previous study [16] evaluated collagen content in pectoralis major in a WB-affected, modern fast-growing broiler strain (Cobb700). A total of 900 birds were raised in 36 pens until d 57. Twenty birds were selected for collagen study in each period of d 35, d 42 and d 57. Selected birds were divided into WB-myopathy-affected and non-affected groups. At d 35, 42 and 57, WB-myopathy broilers showed higher (*p* < 0.05) insoluble collagen content (5.9 vs. 12.1, 6.0 vs. 13.2 and 6.9 vs. 27.4 µg/mg, respectively) in the muscle compared to the non-myopathy broilers. In that same study, micrograph images showed perimysial and endomysial connective tissue spaces filled with collagen tissues in myopathy-affected muscles. Maharjan et al. [16] concluded that when muscle fibers become degenerated, collagen tissues start to fill the space in myopathy-affected broiler muscles. In another study [57], 1125 birds of two modern meat-type strains were raised to d 56 for a collagen and muscle protein turnover study. That study showed a difference in the collagen synthesis rate between strains and a slowdown of the collagen synthesis rate with aging. In that same study, WB-myopathy-affected birds from both strains showed a higher fractional protein degradation rate (FDR) and a lower fractional protein synthesis rate (FSR) during d 42 to d 56 compared to the non-myopathy broilers. Additionally, Maharjan et al. [57] measured the triglyceride synthesis rate in a modern broiler line at d 49. Then, the broilers were divided into WB-myopathy-affected and non-affected groups (n = 10 per group). Triglyceride contents were analyzed in the liver, *Pectoralis major* muscle and fat pad. In non-myopathy broilers, triglycerides accumulated more in the liver (0.20 vs. 0.15%) and fat pad (0.030 vs. 0.005%) compared to the WB-myopathy broilers, whereas in WB-myopathy broilers, triglycerides were highly accumulated in the *Pectoralis major* muscle (0.002 vs. 0.016%) compared to the non-myopathy broilers. Overall, these findings indicate that protein degradation is an important etio-physiological phenomenon of WB-myopathy birds in affected breast muscle tissue, and degraded muscle proteins are replaced by connective tissues like collagen and lipids.

Gene expression studies have indicated that WB-affected muscles exhibit signs of hypoxia, oxidative stress, carbohydrate metabolism dysfunction, and disrupted calcium signaling, all of which contribute to cellular damage. Maharjan et al. [57] showed that downregulated carbohydrate mechanisms, calcium regulation and cell integrity-related genes at d 42 were upregulated at d 56. The birds that were affected by WB myopathy showed downregulated gene expressions of PYGB (carbohydrate metabolism), CSMD3 (cell integrity) and LOC423134 (calcium regulation) at d 42 and showed a response against the conditions by upregulating gene expressions of ALDOB (carbohydrate metabolism), RETSAT (cell integrity) and CXCR4 (calcium regulation) at d 56. This could be indicative of the adaptive response of the muscle fibers in myopathy-affected birds. Oxidative stress and sarcoplasmic reticulum dysfunction may impair calcium regulation. Failed calcium homeostasis can lead to an accumulation of Ca^2+^ within muscle cells, compromising sarcolemmal integrity and triggering myopathic disorders [36,57,58]. In response to oxygen deficiency, the body increases blood flow through nitric oxide production, which inadvertently induces oxidative stress, tissue inflammation, and myodegeneration. This process can result in dilated sarcoplasmic reticulum, mitochondrial swelling, and mitochondrial hyperplasia. Prolonged myodegeneration reduces the regenerative capacity of muscle fibers, ultimately leading to fibrosis and lipidosis, which increases muscle myopathy severity [48,49,54].

## 5. Effect on Meat Quality

Wooden breast, WS, and SM are three major breast myopathies affecting broiler meat quality (Table 2). These conditions alter the physicochemical properties, composition, and overall quality of meat, leading to economic losses and consumer concerns.

WB-affected meat exhibits a reduction in water-holding capacity and an increase in cooking loss and drip loss [7,59,60]. Severe fibrosis in WB meat is associated with reduced lightness and yellowness [42,59]. Additionally, WB meat tends to have a higher ultimate pH, likely due to altered glycolytic and metabolic pathways like reducing glycolysis and TCA cycles [42,53,54]. Fat and collagen content in meat increase, and protein content decreases in WB-affected meat [57,59]. Filets from WB-affected meat are unsuitable for extended freezing due to loss of a significant amount of polyunsaturated fatty acid content, affecting the shelf life of products [61].

**Table 2:** Comparable characteristics of WB-, WS-, and SM-affected meat quality of broilers.

Myopathies	Meat Quality	Sources
Wooden Breast	pH Increased	Chatterjee et al. [60]; Mazzoni et al. [29]; Mudalal et al. [7]
	Lower water holding capacity; higher cooking loss and drip loss	Tijare et al. [31]; Chatterjee et al. [60]; Mazzoni et al. [29]; Mudalal et al. [7]; Cabrol et al. [59]
	Collagen and fat content increased; protein content decreased.	Maharjan et al. [57]; Cabrol et al. [59]; Tasoniero et al. [62]
	Reduced lightness and yellowness	Zambonelli et al. [47]; Cabrol et al. [59]
	Higher compression force with hard, rubbery structure; less succulent	Sun et al. [63]
White Striping	Higher pH	Mudalal et al. [7]; Kuttappan et al. [37]
	Lower water holding capacity; Higher drip loss and cooking loss	Mudalal et al. [7]
	Higher fat, collagen and energy content; lower protein content	Kuttappan et al. [64]
	Higher compression force with lower tenderness and juiciness	Lee et al. [65]
Spaghetti meat	Higher pH	Baldi et al. [5]
	Lower water holding capacity; Higher drip loss and cooking loss	Tasoniero et al. [66]; Soglia et al. [35]
	Lower protein content	Wu et al. [67]
	Higher yellowness	Tasoniero et al. [68]; Wu et al. [67]

In terms of texture, WB-affected meat is characterized by increased compression force in raw conditions. On the other hand, WB-affected meat is more rubbery, less tender, and less succulent when cooked [63]. Compositionally, it contains higher moisture and lipid levels but lower protein content compared to normal breast meat [62].

WS-affected meat shares some similarities with WB. Notably, pH and cooking loss are higher, whereas drip loss is lower in WS meat [7]. The texture is also compromised, with WS meat exhibiting higher compression values, reduced tenderness, and decreased juiciness compared to unaffected meat [65]. In terms of composition, WS meat has higher fat, collagen, and energy content, while protein content is reduced [8].

Among the three myopathies, SM appears to have a lesser impact on meat quality compared to WB and WS [5]. However, SM-affected meat is distinguished by increased yellowness and a higher pH [5,66,67]. Spaghetti meat also has reduced integrity, shear force and protein content [67]. Like WB and WS, SM has reduced water-holding capacity, leading to higher drip loss and cooking loss [38,68]. This reduction in water retention is primarily attributed to lower protein solubility caused by muscle degradation [66].

These myopathies can occur individually or simultaneously, with WB being the most severe, followed by WS and SM [32,34]. Among them, WS has the closest meat quality to unaffected meat, while WB alone or in combination with SM causes the most detrimental effects on breast meat quality. Given their significant impact on meat quality, further research is crucial to identifying and preventing WB and WS in broilers [69].

## 6. Detection and Diagnosis

Broiler breast myopathies, particularly white striping (WS) and woody breast (WB), have become increasingly prevalent in poultry production, necessitating the development of effective detection methods. These myopathies are commonly identified through subjective visual and tactile evaluations [31,60]. However, this approach presents challenges, including labor intensity and time consumption, which limit its efficiency in high-throughput processing environments.

### 6.1. Subjective Methods

Subjective methods generally rely on tactile assessments, visual inspection, and macroscopic evaluations. For instance, Sihvo et al. [3] proposed a palpation method to assess the hardness of WB-affected filets, alongside macroscopic evaluations such as color, shape, and the presence of exudates or hemorrhages. To standardize this method, Tijare et al. [31] developed a 4-point scoring scale for palpation, ranging from 0 (normal) to 3 (severe). However, tactile scoring requires expertise and must be conducted under consistent temperature, light, and humidity conditions [66]. Additionally, distinguishing between WB and WS using visual cues alone has proven to be challenging [34]. Measuring compression force has been a means to objectively quantify hardness and is highly correlated with hand palpation scores [63]. To address these limitations, some researchers have turned to digital tools. Digital palpation devices, for example, have been studied and are more consistent for objective WB scoring [66].

### 6.2. Objective Methods

While subjective methods are relatively low-cost and require minimal machinery, they remain laborious, time-consuming, and impractical for large-scale industrial applications. Therefore, there is a pressing need for more efficient objective methods. Objective detection methods for broiler breast myopathies include instrumental texture analysis, gene expression profiling, and metabolomic studies [7,8,47,55]. While these methods provide valuable insights, they are labor-intensive and slow, often requiring tissue, cell, or blood samples, which makes them more suitable for research rather than industrial use.

### 6.3. Emerging Methods

Emerging technologies have shown promise for faster, more efficient detection. Computer vision systems (CVS) and optical coherence tomography (OCT) have been explored for detecting breast myopathies [70], although OCT’s effectiveness is limited [71]. Near-infrared spectroscopy (NIR) is considered one of the most promising objective methods, especially for industrial production lines, as it can detect WB by measuring muscle protein content and water-binding conditions [70]. However, the results can vary depending on the layer of breast being tested [72]. Combining NIR with CVS could improve detection accuracy, as suggested by Geronimo et al. [70]. In addition to these methods, newer techniques such as dielectric spectroscopy (DS) and time-domain nuclear magnetic resonance (TD-NMR) have been explored for detecting WB [73]. Structured-illumination imaging coupled with surface profilometry is another promising development for breast myopathy detection [74].

While the focus on WB and WS has grown in recent years, other myopathies, such as SM, continue to be largely diagnosed through visual screening, sometimes supplemented by transcriptomic profiling and biomarker analysis [75]. Interestingly, WS has not yet been included in the USDA’s official poultry product grading system [46], which may explain the relative lack of research on this condition. WS is typically characterized by white striations in the breast muscle, which can be detected through visual evaluation. A scoring system developed by Kuttappan et al. [8] classifies the severity of WS based on striation thickness, ranging from 0 (normal) to 3 (severe). Additionally, histopathological analyses reveal associated changes, including necrosis, atrophy, macrophage infiltration, and fat accumulation [5]. Recent studies have also linked gene expression markers such as MuRF-1, MYOZ2, PPARγ, Insulin Receptor (IR) and GPx7, as well as upregulated proteins like LIPE, UCP1, ATP5IF1, and DMD, with the severity of WS [44,75].

Overall, while subjective methods remain prevalent for detecting broiler breast myopathies, objective techniques, including those based on instrumental analysis, digital technologies, and molecular profiling, are rapidly emerging. Continued research and development of these methods will be critical for improving detection efficiency, accuracy, and applicability in both research and industrial settings.

## 7. Strategies for Mitigation and Prevention

Breast pectoral myopathies, though linked to growth and weight gain in broilers, have prompted various nutritional interventions aimed at preventing or reducing their severity. One of the primary approaches has been feed restriction. Most studies on feed restriction have applied 80%, 85%, 90%, and 95% feed limitations during early growth stages (days 12–18) [76]. While feed restriction was found to reduce the occurrence of WB and WS, it did so at the cost of growth performance and yield. In addition, nutrient restriction, such as amino acid, energy, or protein limitation, and supplementation with antioxidants and feed additives that have antioxidative and anti-inflammatory effects were also found to be effective to varying degrees in minimizing broiler breast myopathies.

### 7.1. Nutrient Restrictions

Another key area of focus has been the manipulation of crude protein (CP) and energy levels in broiler diets. Abreu et al. [77] reported no relationship between CP levels (19% vs. 21%) and breast myopathies, while Johnson et al. [78] found no association between varying energy levels (3062 vs. 3172 kcal/kg) or digestible lysine levels (0.99 vs. 1.07) and the incidence of WS or WB. However, a previous study by Kindlein et al. [79] using different dietary densities (3300 kcal ME/kg and 19.56% CP vs. 3050 kcal ME/kg and 18.37% CP) observed a reduction in the incidence and severity of WS in broilers fed the lower-density diet. Similarly, Meloche et al. [76] tested diets with 85% and 75% of lysine requirements during the early (12–18 days) and later (19–26 days) stages, finding that 85% lysine supplementation reduced the incidence of WB and WS without affecting final body weight. Cruz et al. [80] also showed that diets with 63% and 70% digestible lysine reduced WB and WS incidence but negatively impacted growth performance and breast yield.

In a study by Bertechini et al. [81], different energy levels, lysine sources, and calcium pidolate supplementation were evaluated for their effect on WB and WS. They found that lysine from Lys-SO_4_ compared to Lys-HCl and calcium pidolate supplementation similarly reduced WB and WS. Bodle et al. [82] tested various strategies, including an increased arginine-to-digestible-lysine ratio, 94.4 mg/kg vitamin C supplementation, twice-daily supplementation of vitamin premix, and a 15% reduction in digestible amino acids during the grower phase. Among these, increasing the arginine-to-digestible-lysine ratio, vitamin C supplementation, and reducing amino acid levels by 15% were found to reduce the incidence of WB without negatively affecting growth performance. The increased arginine in the diet enhances nutrient and metabolite exchange in muscle tissue, promoting muscle fiber hypertrophy [82]. Vitamin C functions as an antioxidant, mitigating hypoxia to reduce muscle myopathies, while a reduction in amino acids during the growth phase supports satellite cell growth and muscle fiber recovery in later stages [83]. In contrast, Livingston et al. [84] reported that a combination of glutamine and arginine led to an increased incidence of WB and WS, likely due to higher glutamine levels.

The findings suggest that moderate and strategically timed nutrient restriction, particularly of digestible lysine, total amino acids, and dietary energy density, can reduce the incidence and severity of white striping (WS) and wooden breast (WB) in broilers. The beneficial effects appear to be connected to controlled muscle growth, improved vascularization, and reduced metabolic and oxidative stress in rapidly developing breast muscle. However, excessive restriction may compromise growth performance and breast yield, indicating that a careful balance is essential.

### 7.2. Antioxidant Supplementation

Given that hypoxia is a primary cause of myopathy, antioxidant supplementation has also been explored as a potential remedy. Several studies have tested vitamin E at varying levels (from 15 to 400 IU/kg) but found no significant effect on the incidence of myopathies, possibly due to the inability of vitamin E to reach muscle cells effectively because of an underdeveloped capillary system [84]. On the other hand, Wang et al. [85] supplemented broiler diets with DL-α-tocopherol acetate (200 IU/kg) and omega-3 fatty acids, observing a reduction in WB incidence, which highlighted DL-α-tocopherol acetate as a more effective form of vitamin E, likely due to its ability to reach cells where hypoxia occurs [86]. In contrast, selenium supplementation, such as zinc-L-selenomethionine, did not reduce breast myopathies [87]. Kuttappan et al. [6] found that a mix of chelated minerals (zinc, copper, manganese, and selenium sulfate), alone or in combination with the antioxidant ethoxyquin, was effective against WB incidence in broilers.

### 7.3. Feed Additives Supplementation

Feed additives have also been investigated for their potential in mitigating breast myopathies. Maynard et al. [88] found that supplementation with 0.12% guanidinoacetic acid, a creatine precursor, was effective against pectoral myopathies. Similarly, De Souza et al. [89] showed that 0.06% guanidinoacetic acid supplementation reduced the incidence of breast myopathies, and Dayan et al. [90] reported reduced SM incidence in broilers supplemented with 0.06% guanidinoacetic acid. Guanidinoactic acid works as a precursor of creatine, which increases vimentin protein in the muscle that maintains the sarcomere cytostructure of muscle fibrils. Additionally, Greene et al. [91] demonstrated that supplementation with 1000 FTU/kg and 2000 FTU/kg phytase reduced WB myopathy by 5%. A similar effect was observed in another study using 4^th^-generation phytase at 2000 FTU/kg [92]. The mechanism of phytase in reducing myopathy is still unknown. However, Greene et al. [93] linked phytase with better nutrient digestibility and bioavailability of calcium and phosphorus. Probiotics, however, were found to be ineffective in reducing breast myopathies [94]. In contrast, supplementation with 400–1200 mg/kg hydrolyzed yeast was effective in reducing WB in broilers [95]. The redox balance capacity of yeast might be a reason for reducing WB occurrence and severity. Similarly, Lee et al. [96] found that 0.4% chitosan supplementation partially reduced WS damage in broilers, while Tesser et al. [97] reported that 0.5 mg and 1 mg/kg of chromium-methionine effectively reduced WB incidence in heat-stressed broilers. The effect is attributed to chitosan and chromium-methionine’s antioxidative capacities and their ability to reduce lipid metabolism in muscle.

## 8. Conclusions

Modern broiler breast myopathies like WB, WS, and SM have emerged as major quality defects accompanying the rapid genetic progress and production efficiency achieved in the poultry industry. This review highlights that these conditions are multifactorial disorders closely associated with accelerated muscle growth, altered muscle fiber structure, metabolic stress, and impaired vascularization within the pectoralis major muscle. The results of hypoxia, oxidative stress, inflammation, and degeneration ultimately lead to the structural and compositional changes observed in affected breast meat.

Substantial progress has been made in characterizing the histological, biochemical, and physiological alterations associated with these myopathies. However, the exact sequence of events leading from rapid muscle hypertrophy to muscle degeneration is still not completely understood. Current evidence suggests that the imbalance between muscle growth rate and the development of adequate vascular and connective tissue support plays a central role in the onset of these disorders.

Reliable and rapid detection methods remain essential for both research and industry applications. Advances in imaging technologies, meat quality assessment tools, and machine-learning-based classification systems show promise for improving the early identification and classification of affected filets in commercial processing environments.

Various mitigation strategies have been explored, including genetic selection, nutritional interventions, management adjustments, and the use of functional feed additives such as antioxidants and metabolic modulators. While some nutritional strategies, such as controlled growth rates, optimized amino acid supply, and antioxidant supplementation, have shown potential to reduce the severity or incidence of myopathies, these approaches must be carefully balanced to avoid compromising broiler growth performance and breast meat yield.

Future research should focus on integrating genetics, nutrition, physiology, and management to better understand the complex biological mechanisms underlying these disorders. A multidisciplinary approach combining molecular biology, muscle physiology, and precision nutrition will be critical for developing effective prevention strategies. Ultimately, improving our understanding of modern broiler myopathies will help sustain broiler production efficiency while maintaining meat quality and economic profitability for the poultry industry.

## Figures and Tables

**Table 1:** Changes in broiler performance and breast meat parameters between 1957 and 2024.

Parameters	Before 1980	2000–2005	2018–2024	Sources
Weight at day 0, g	34	44	44	Havenstein et al. [10]; Zuidhof et al. [11], Aviagen [12]
Weight on day 56, g	905	4202	4318	
FCR day 56	2.84	1.918	1.793	
Breast meat, %	9.7% on day 43	22.2% on day 32	23.4% on day 32	Havenstein et al. [10]; Grashorn et al. [13]; Aviagen [12]
Pectoralis major fiber diameter, µm	18	29	46	Kikuchi et al. [14]; Guarnier et al. [15]; Maharjan et al. [16];
Density of myofiber (number/mm^2^)	-	250	642	Rehman et al. [17] Scheuermann et al. [18]

## Data Availability

No new data were created or analyzed in this study.
